# Monitoring Unfractionated Heparin in Adult Patients Undergoing Extracorporeal Membrane Oxygenation (ECMO): ACT, APTT, or ANTI-XA?

**DOI:** 10.1155/2021/5579936

**Published:** 2021-05-03

**Authors:** Tung Phi Nguyen, Xuan Thi Phan, Dai Quang Huynh, Ha Thi Viet Truong, Yen Nguyen Hai Le, Tuan Manh Nguyen, Quan Quoc Minh Du, Thao Phuong Le, Hai Ngoc Truong, Thi Thi Ho, Thao Thi Ngoc Pham

**Affiliations:** ^1^Department of Intensive Care, Ho Chi Minh City University of Medicine and Pharmacy, Ho Chi Minh City, Vietnam; ^2^Intensive Care Unit, Vinmec International Hospital, Hanoi, Vietnam; ^3^Intensive Care Unit, Cho Ray Hospital, Ho Chi Minh City, Vietnam

## Abstract

**Background:**

During ECMO, anticoagulants, in particular, unfractionated heparin (UFH), are commonly used and monitored by laboratory tests, including ACT, APTT, and anti-Xa level.

**Method:**

A single-center retrospective observational study was conducted on adult patients undergoing ECMO between January 2019 and January 2020 at a tertiary hospital. The correlations between ACT, APTT, anti-Xa, antithrombin, and UFH dose were assessed.

**Results:**

129 sets of measurements from 37 patients were obtained including ACT, APTT, anti-Xa, antithrombin, and UFH dose measured simultaneously. 102 out of 129 sets of values were interpreted as antithrombin deficiencies. The correlation coefficient between APTT and anti-Xa; ACT and anti-Xa are 0.72 and 0.33, respectively, *p* < 0.001. The patients with normal antithrombin levels exhibited a significant correlation between APTT and anti-Xa (*r* = 0.80, *p* < 0.001). ACT, on the other hand, was poorly correlated with UFH dose, whether there is AT deficiency or not. Anti-Xa and APTT are only moderately correlated with UFH dose in the group without antithrombin deficiency, with correlation coefficients of 0.62 and 0.57, respectively, *p* < 0.05.

**Conclusion:**

APTT value is strongly correlated with anti-Xa value, particularly in patients with normal antithrombin levels. However, the ACT value was poorly correlated with anti-Xa and not with the UFH dose. In groups without antithrombin deficiency, APTT and anti-Xa values only moderately correlated with UFH dose.

## 1. Background

Extracorporeal membrane oxygenation (ECMO) has been widely accepted as a treatment for life-threatening cardiac and pulmonary failure [1, 2]. As with any other artificial system, coagulation will be activated during ECMO as a consequence of shear stress and exposure of blood to the nonbiologic surfaces of the ECMO circuit [3–6]. Heparin indirectly inhibits thrombin activity by binding to a specific active site of the enzyme inhibitor antithrombin (antithrombin III, AT), causing a conformational change that results in increasing its anticoagulant activity 1000-fold to 4000-fold [7]. Heparin resistance in patients undergoing ECMO is often a multifactorial phenomenon in which acquired AT deficiency is partly attributed [4]. Nevertheless, a proportion of potentially active heparin is reversibly bound and neutralized by plasma protein, so-called heparin-binding proteins, which are acute phase reactants found in critically ill patients [8, 9].

The efficacy of UFH is monitored by ACT, APTT, and anti-Xa tests. The ACT is a whole-blood-based point-of-care test (POCT) that measures the rate of clot formation. Therefore, an ACT result can be prolonged by multiple factors independent of UFH doses, such as anemia, hypofibrinogenemia, thrombocytopenia, other than heparin inhibited coagulation factor deficiencies, hypothermia, and hemodilution [5, 10]. Since its first description in 1953, Activated Partial Thromboplastin Time (APTT) is frequently used to titrate heparin dose outside of the operating room [11]. APTT test, using platelet-poor plasma from centrifuged whole blood, is measured by photo-optical method or magnetic steel ball method (each has different strengths and weaknesses). The APTT target range is varied, depending on the method utilized and the various reagents used. Also, its value is influenced by comorbidities such as vitamin K deficiency, liver disease or hemodilution, other inherited factor deficiencies such as vitamin K deficiency, liver disease or hemodilution, other inherited factor deficiencies such as hemophilia or lupus anticoagulant-type inhibitors [12]. However, cases of inherited coagulopathy or autoimmune disease are not candidates of ECMO. In 1973, Denson and colleagues introduced anti-Xa—which does not measure the heparin concentration in blood but estimates the anticoagulant effect of heparin by measuring the concentration of antithrombin-heparin complex. This complex reflects an exact concentration and the effectiveness of heparin in patients with normal antithrombin levels [4, 11, 13]. In contrast to the ACT and aPTT, the anti-Xa assay is specific to the anticoagulant effect of UFH and is not influenced by coagulopathy, thrombocytopenia, or dilution [5]. However, an anti-Xa value is also affected by technical errors from the photo-optical method, such as hyperbilirubinemia, hemolysis, and antithrombin deficiency [4, 13].

Until now, there is no complete consensus on anticoagulation strategy and its monitoring during ECMO support [4, 5]. Unfortunately, an anti-Xa assay is frequently not feasible since it is not widely available and difficult to perform several times a day and longer waiting times than ACT and aPTT. Therefore, we conducted an observational study to assess the correlations between ACT, APTT, anti-Xa, and administered UFH dose on adult ECMO patients.

## 2. Methods

### 2.1. Research Design

We retrospectively conducted an observational study of adult patients (age ≥18 years) who received peripheral ECMO in the Intensive Care Unit (ICU) of Cho Ray Hospital (a tertiary hospital) between January 2019 and January 2020. This study was approved by the Research Ethics Committee of Ho Chi Minh City University of Medicine and Pharmacy (IRB-VN01002) and by the Research Ethics Committee of Cho Ray Hospital (No.122/HĐĐĐ).

### 2.2. Patient Selection

#### 2.2.1. Inclusion Criteria

All adult patients (age ≥18 years) who received ECMO in the Intensive Care Unit (ICU) of Cho Ray hospital between January 2019 and January 2020 with laboratory results of ACT, APTT, anti-Xa, antithrombin tests, and simultaneous UFH dosage were included.

#### 2.2.2. Exclusion Criteria

Patients who underwent ECMO for less than 24 hours, patients with pulmonary embolism who were treated with fibrinolytic therapy before or during ECMO.

### 2.3. Clinical Data

Baseline characteristics included patients' demographics (age, gender), diagnosis at ICU admission, ECMO indication, and ECMO types. Major bleeding complications were defined by the ELSO Anticoagulation guideline [5], and hospital mortality was also recorded.

To accurately examine the correlation of UFH monitoring tests, only patients who had ACT, APTT, anti-Xa, and antithrombin tests performed simultaneously with the blood samples were taken at the same time. UFH dosage (units per hour) at the time of obtaining the blood sample was also recorded. Any case with missing or incorrectly recorded results was eliminated from the research. Patients undergoing ECMO for more than 14 days had data recorded up to 14 days to be representative of the study population. Antithrombin activity level of less than 80 percent of the reference range in our laboratory was defined as antithrombin deficiency.

### 2.4. ECMO Configuration

Our department used the ECMO system, which includes a Rotaflow console, PLS membrane, and cannulas from Maquet, Getinge group, Sweden.

### 2.5. ECMO Anticoagulation Protocol

In our center, the anticoagulation protocol was implemented based on the ELSO guideline. Patients received an initial UFH bolus dose of 50–100 units per kilogram of body weight at the time of cannulation for ECMO, and then the dose was maintained as a continuous infusion during the ECMO course. The UFH dose was adjusted based on clinical factors such as the risk of hemorrhage and thrombosis, including current evidence of hemorrhage, coagulation tests, and underlying diseases. The UFH infusion was initiated at a dose of 7.5–20 UI/kg per hour. Assessment of the activity of UFH would likely rely on three therapeutic monitoring blood tests: ACT and APTT at every 6 hours and anti-Xa at every 24 hours. Anti-Xa was only available on weekdays. Therapeutic goals were defined as an ACT of 180–220 seconds (local reference range 90–130 sec), an APTT of 45–80 seconds (local reference range 25.1–36.5 sec), and an anti-Xa level of 0.3–0.7 UI/mL (local reference range 0.00 UI/ml). Since there is no specific UFH monitoring protocol, the physician will decide to adjust the UFH dose according to the risk of bleeding and the risk of thrombosis at that time. Plasma was infused in the case of severe antithrombin deficiency (antithrombin <50%) and/or heparin resistance.

### 2.6. Laboratory Measurement

ACT assay was conducted by Medtronic Activated Clotting Time Cartridges device, including 2 kits which are LR-ACT (ACT low range) and HR-ACT (ACT high range).

APTT assay was performed on 3 devices: STA-R Evolution (kaolin as activator, magnetic method); ACL TOP 750 (SynthAsil reagent, photo-optical method); SYSMEX 2500 (ACTIN FSL reagent, photo-optical method). In the case of hyperbilirubinemia or hemolysis, the magnetic method was preferred.

Anti-Xa was performed on ACL TOP 750 device, photo-optical method, without exogenous antithrombin. Blood was collected in a tube with a citrate anticoagulant. Platelet-poor plasma is isolated from the whole blood using centrifugation at 4000 RPM for 5 minutes on ROTINA 380 machine. Platelet-Poor Plasma was put into an anti-Xa test kit on ACT TOP 750 machine with 2.5 ml of factor-Xa premixed. Reference value: Normal: 0.0 IU/ml; Limit of results 0.0–2 IU/ml.

### 2.7. Statistical Analysis

Data were expressed as percentages, median (interquartile range, IQR), or mean ± standard deviation (SD). Student's *t* test was used for quantitative variables with normal distribution or Wilcoxon rank-sum test in case of nonnormal distribution in 2 groups: reduction in antithrombin and nonreduction in antithrombin. Pearson/Spearman correlation coefficients and scatter plots were used to examined and presented for visual assessment of the association. A *p* value of less than 0.05 was considered statistically significant.

## 3. Results

During the study period, 37 patients were enrolled and 129 tests of measurement (including ACT, APTT, anti-Xa, antithrombin, and UFH dose) were obtained (Table 1).

The average age of these patients was 40 (IQR 32–50), and there were no statistically significant differences in gender. The most common indications for ECMO were acute myocarditis (54.0%), ARDS (35.1%), myocardial infarction (8.1%), and severe anaphylaxis (2.7%). There were 23 VA-ECMO (62.1%), 13 VV-ECMO (35.1%), and 1 VAV-ECMO. The median ECMO duration was 7 days (IQR 4–12 days).

The incidence of antithrombin deficiency was 79% (102/129 kits). In comparison with the group without antithrombin deficiency, the antithrombin deficiency group displayed higher ACT results and UFH dose, but not anti-Xa value (Table 1).

**Figure 1:**
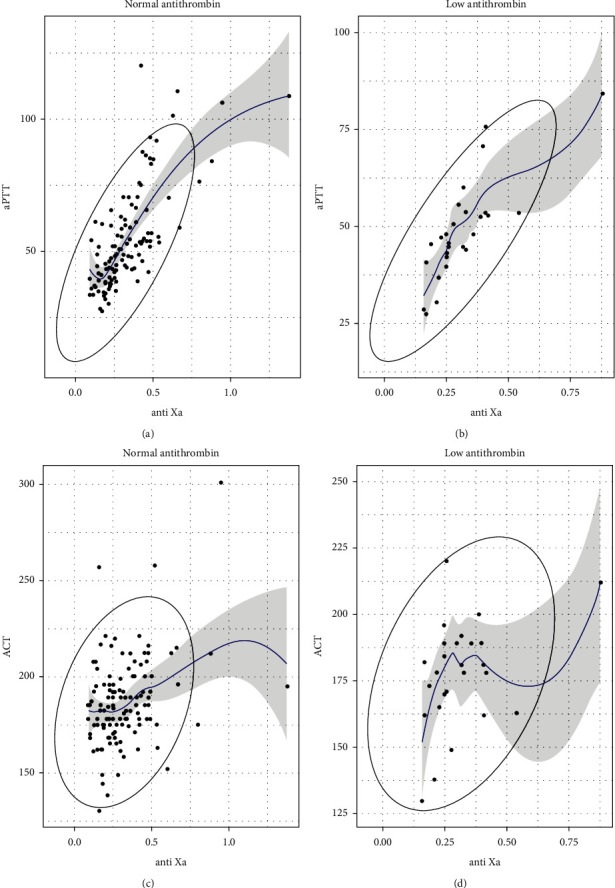
Scatter plot showing correlation between APTT, ACT, and anti-Xa.

The study showed a strong correlation between APTT and anti-Xa values with a correlation coefficient of 0.72. This correlation was stronger when the patients were in the group with antithrombin deficiency, which was shown in Figure 1(a): the linear regression line of the scatter plot went up following the direction of the short axis of the eclipse. While ACT values had a weak positive correlation with anti-Xa values in both groups with and without antithrombin deficiency, the trend line, illustrated on the Figure 1(b), went up but still had wide fluctuation, and the 2 axes of the eclipse almost had the same length.

**Figure 2:**
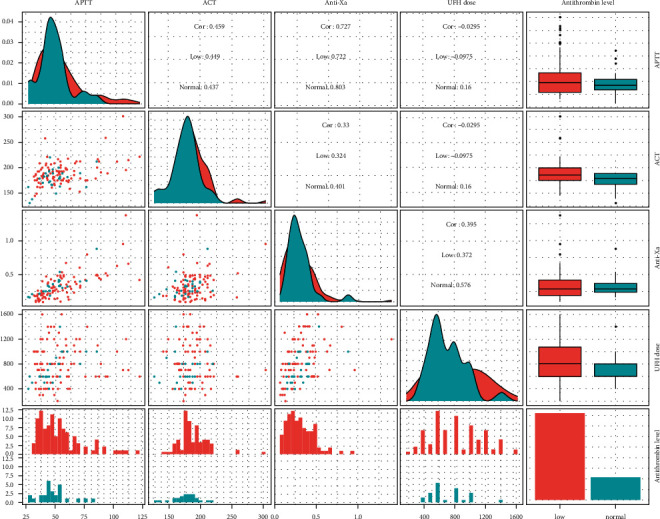
Correlation between ACT, APTT, anti-Xa and UFH dose values based on antithrombin groups.

For UFH dose at that time, the only anti-Xa value had a moderate positive correlation with UFH dose, while APTT value had a moderate positive correlation with UFH dosing and ACT had no correlation with UFH dosing of patients in both groups with and without antithrombin deficiency. All the correlations are shown in Table 2 and demonstrated in Figure 2.

## 4. Discussion

UFH is monitored by ACT, APTT, and anti-Xa. Of the three tests above, anti-Xa is considered to be more reliable for heparin concentration than the other two [5]. However, anti-Xa is not as commonly ordered or as often (many times a day) due to its high cost and complicating techniques. ACT is considered the most common test being a fast and inexpensive method despite its limited reliability [3, 5]. The value of the APTT test in monitoring UFH in ECMO has recently gained more attention. In recent studies, APTT moderately correlated with anti-Xa, UFH dose, and UFH concentration, while ACT poorly correlated with anti-Xa, UFH dosing, and UFH concentration [11, 14–17]. These researches were mainly done in pediatric ECMO patients. Most of the reagents for the anti-Xa assay require a patient's endogenous antithrombin instead of adding exogenous antithrombin because the prevalence of antithrombin deficiency in the general population is very low. However, acquired antithrombin deficiency has been associated with patients on ECMO, which results in a lower value of anti-Xa than heparin concentration. Therefore, we investigated the subgroup of patients without antithrombin deficiency when the anti-Xa value correlates better with heparin concentration in order to reappraise the value of ACT and APTT tests.

In our study, there was a strong positive relationship between the value of APTT and the value of anti-Xa (*r* = 0.72, *p* < 0.001), especially in the normal antithrombin level group (*r* = 0.80, *p* < 0.001). Meanwhile, ACT value correlated poorly with anti-Xa, even in the subgroup of patients with normal antithrombin level. Khaja et al. also found that APTT value correlated closer with anti-Xa than ACT value (*r* = 0.364 vs. 0.125, respectively). However, this study was conducted on infants with a small sample size (21 patients) and no analysis on the role of antithrombin. In another study by Liveris et al., 17 pediatric and neonatal ECMO patients had shown similar results; the correlation of coagulation monitoring assay with UFH dose was poor with ACT assay, moderate with APTT assay, and strong with anti-Xa assay [11]. There have been few studies to evaluate the correlation of ACT, APTT, anti-Xa, and heparin dose in adult ECMO patients. One research by Atallah et al. on 46 patients receiving ECMO found a Pearson correlation of APTT and heparin dose from 0.43 to 0.54; meanwhile, the Pearson correlation of ACT was only 0.11–0.14 [14]. However, the UFH dose cannot represent the efficacy or concentration of UFH in adult patients, especially in critically ill patients and ECMO patients.

The ACT is a whole-blood test. Therefore it is influenced by all of the properties in the hemostasis system rather than only being effective to UFH. ACT is also prone to technical errors. Within the results of this study, we showed that when taking antithrombin level into consideration, ACT in the group without antithrombin deficiency was statistically higher. Unlike ACT, the APTT value correlated strongly with anti-Xa at the same time. Besides the fact that APTT is a more reliable tool to monitor anticoagulatory activity, APTT assay in our study was also done by both photo-optical and magnetic measurement methods. This helped to minimize the weaknesses of the test in cases of increased bilirubin or hemolysis and increased the sensitivity of the test.

Regarding the UFH dose, it is usually only maintained within the therapeutic range, so no outlier value was observed. In patients without antithrombin deficiency, APTT and anti-Xa values were strongly and positively correlated with each other and with UFH dose. Therefore, the UFH dose was lower and less fluctuating. For patients with antithrombin deficiency, ACT, APTT, and anti-Xa values were lower and did not correlate well with each other, causing a higher and unsteady UFH dose.

The results of this study have shown that APTT is more reliable than ACT in monitoring the efficacy and titrating UFH dose in adult patients on ECMO. We also found that the value of APTT in monitoring UFH was no less than the anti-Xa level for the following reasons: (1) In patients without antithrombin deficiency, APTT strong correlation with anti-Xa; (2) in patients with antithrombin deficiency, both APTT and anti-Xa did not correlate well with UFH dose; (3) APTT is common and can be performed several times a day for monitoring UFH. In the future, APTT should be reaccessed in parallel with a more precise measuring method than the traditional quantitative anti-Xa method, such as measurement of anti-Xa by adding exogenous antithrombin in patients with antithrombin deficiency.

### 4.1. Strengths and Limitations

To our knowledge, this is the first study that was conducted on adult ECMO patients to assess ACT, APTT, anti-Xa, and UFH dose regarding antithrombin level, to determine the relationship of these tests in a different perspective.

Several limitations might be taken into account when considering our results. Firstly, this is a single-center study, so our results might have limitations when trying to generalize for other ICUs or other settings. Secondly, the sample size was small. This promotes further studies in the future.

## 5. Conclusion

In adult patients undergoing ECMO using UFH anticoagulants, there was a strong positive correlation between the value of APTT and the value of anti-Xa, with an especially strong correlation in patients with normal antithrombin levels. However, the ACT value was poorly correlated with anti-Xa and did not correlate with UFH dose. Values such as APTT and anti-Xa only correlate moderately with heparin dose in groups without antithrombin deficiency.

## Figures and Tables

**Table 1:** ACT, APTT, anti-Xa, and UFH dose values.

	Total (*N* = 129)	Group without antithrombin deficiency (*n* = 27)	Group with antithrombin deficiency (*n* = 102)	*p* value
ACT (second)	184 [175; 196]	178 [168; 189]	185 [175; 200]	0.033
APTT (second)	49 [40; 59]	47 [42; 54]	50 [40; 60]	0.300
Anti-Xa (IU/ml)	0,28 [0.21; 0.41]	0,28 [0.24; 0.37]	0.28 [0.19; 0.42]	0.812
Heparin dose (IU/h)	800 [600; 1000]	600 [600; 800]	800 [600; 1075]	0.110

Data are presented as median [interquartile range].

**Table 2:** Correlation between ACT, APTT, anti-Xa values, and concurrent heparin dosing.

Correlation coefficient spearman	All	Normal antithrombin level
APTT vs. anti-Xa	0.72 (*p* < 0.001)	0.80 (*p* < 0.001)
ACT vs. anti-Xa	0.33 (*p* < 0.001)	0.40 (*p*=0.038)
APTT vs. UFH dose	0.14 (*p* < 0.100)	0.62 (*p* < 0.001)
ACT vs. UFH dose	−0.03 (*p* < 0.740)	0.16 (*p*=0.420)
Anti-Xa vs. UFH dose	0.39 (*p* < 0.001)	0.57 (*p*=0.001)

## Data Availability

Data are available upon request to the authors. A summary of relevant information will be published with the manuscript.
